# Left ventricular function and coronary microcirculation in patients with mild reduced ejection fraction after STEMI

**DOI:** 10.1186/s12872-022-02846-9

**Published:** 2022-09-25

**Authors:** Yuliang Ma, Lan Wang, Wenying Jin, Tiangang Zhu, Jian Liu, Hong Zhao, Jing Wang, Mingyu Lu, Chengfu Cao, Bailin Jiang

**Affiliations:** 1 Beijing Key Laboratory of Early Prediction and Intervention of Acute Myocardial Infarction; Center for Cardiovascular Translational Research, Department of Cardiology, Peking University People’s Hospital, Beijing, China; 2 Department of Anesthesiology and Pain Medicine, Peking University People’s Hospital, Beijing, China

**Keywords:** Acute ST-segment elevation myocardial infarction, Heart failure, Ejection fraction, Speckle tracking imaging, Myocardial contrast echocardiography, Coronary microcirculation

## Abstract

**Background:**

The characteristics of heart failure (HF) with mildly reduced ejection fraction (EF) (HFmrEF) overlap with those of HF with reduced EF (HFrEF) and HF with preserved EF (HFpEF) and need to be further explored. This study aimed to evaluate left ventricular (LV) function and coronary microcirculation in patients with mildly reduced ejection fraction after acute ST-segment elevation myocardial infarction (STEMI).

**Methods:**

We enrolled 119 patients with STEMI who had undergone speckle tracking imaging and myocardial contrast echocardiography during hospitalization from June 2016 to June 2021. They were classified into normal, HFmrEF, and HFrEF groups according to their left ventricular EF (LVEF): ≥ 50%, 40–50%, and ≤ 40%, respectively. The data of the HFmrEF group were analyzed and compared with those of the normal and HFrEF groups.

**Results:**

HFmrEF was observed in 32 patients (26.9%), HFrEF in 17 (14.3%), and normal LVEF in 70 patients (58.8%). The mean global longitudinal strain (GLS) of all patients was − 11.9 ± 3.8%. The GLS of HFmrEF patients was not significantly different from that of the HFrEF group (− 9.9 ± 2.5% and − 8.0 ± 2.3%, respectively, *P* = 0.052), but they were both lower than that of the normal group (− 13.8% ± 3.5%, *P* < 0.001). The HFmrEF group exhibited significantly poorer myocardial perfusion index (1.24 ± 0.33) than the normal group (1.08 ± 0.14, *P* = 0.005) but displayed no significant difference from the HFrEF group (1.18 ± 0.19, *P* = 0.486). Moreover, a significant difference in the incidence of regional wall motion (WM) abnormalities in the three groups was observed (*P* = 0.009), and the WM score index of patients with HFmrEF was 1.76 ± 0.30, similar to that of patients with HFrEF (1.81 ± 0.43, *P* = 0.618), but poorer than that in the normal group (1.33 ± 0.25, *P* < 0.001).

**Conclusions:**

GLS is a more sensitive tool than LVEF for detecting LV systolic dysfunction. The LV systolic function, coronary microcirculation, and WM in patients with HFmrEF was poorer than that of patients with normal LVEF, but comparable to that in patients with HFrEF. Patients with HFmrEF after STEMI require more attention and appropriate management.

## Background

Heart failure (HF) has been classified into HF with reduced ejection fraction (EF) (HFrEF) and HF with preserved EF (HFpEF) according to left ventricular EF (LVEF) [1–3]. However, heterogeneity in the response of patients with HFpEF to certain drugs has been observed [4–6]. Meanwhile, echocardiographic measurement cannot reliably distinguish an LVEF change of < 10%. Therefore, a buffer zone between HFrEF and HFpEF meant that misclassification would be less likely [7]. Thus, in 2016, the European Society of Cardiology (ESC) introduced the concept of HF with mid-range EF in acknowledgment of the ‘gray area’ between HFrEF and HFpEF and to improve identification of the latter [2]. In 2021, the ESC Universal Definition and Classification of HF proposed that HF with LVEF of 40–49% be termed HF with mildly reduced EF (HFmrEF) [8]. The characteristics of HFmrEF overlap with those of HFrEF and HFpEF. Patients in this range may have etiologies that are similar to those of patients with HFrEF or HFpEF, and may transition from higher to lower LVEF or vice versa. The recognition of HFmrEF is gradually evolving.

Speckle tracking imaging (STI)-derived global longitudinal strain (GLS) is a reproducible and feasible parameter in clinical use, that offers incremental prognostic data over LVEF in a variety of cardiac conditions [9]. Myocardial contrast echocardiography (MCE) has been used to evaluate resting microvascular flow following the emergency management of ST-segment elevation myocardial infarction (STEMI) [10]. Persistent resting coronary microcirculation dysfunction (CMD) has been shown to provide independent predictive value regarding adverse left ventricular (LV) remodeling and recurrent cardiac events [11, 12]. Therefore, this study aimed to examine LV function and coronary microcirculation in patients with mildly reduced LVEF with STEMI.

## Methods

### Subjects

All consecutive patients with STEMI who had completed STI and MCE during hospitalization at Peking University People’s Hospital from June 2016 to June 2021 were enrolled in the study. Patients were diagnosed with STEMI after an expert physician review based on the 2018 Joint ESC/American College of Cardiology/American Heart Association/World Heart Federation fourth universal definition of myocardial infarction [13]. Patients who did not complete STI or MCE during hospitalization were excluded from the study during the initial stage. Patients were subsequently categorized into the normal group (LVEF ≥ 50%), HFmrEF group (LVEF 40–50%), and the HFrEF group (LVEF ≤ 40%). STI and MCE data of the HFmrEF group were analyzed and compared with those of the normal and HFrEF groups. Demographic parameters including age, sex, body mass index (BMI), clinical characteristics including history of smoking, hypertension, diabetes mellitus, chronic kidney disease, atrial flutter/fibrillation, laboratory data, coronary angiography data, and revascularization data were extracted.

### Echocardiogram, STI and MCE

**Fig. 1:**
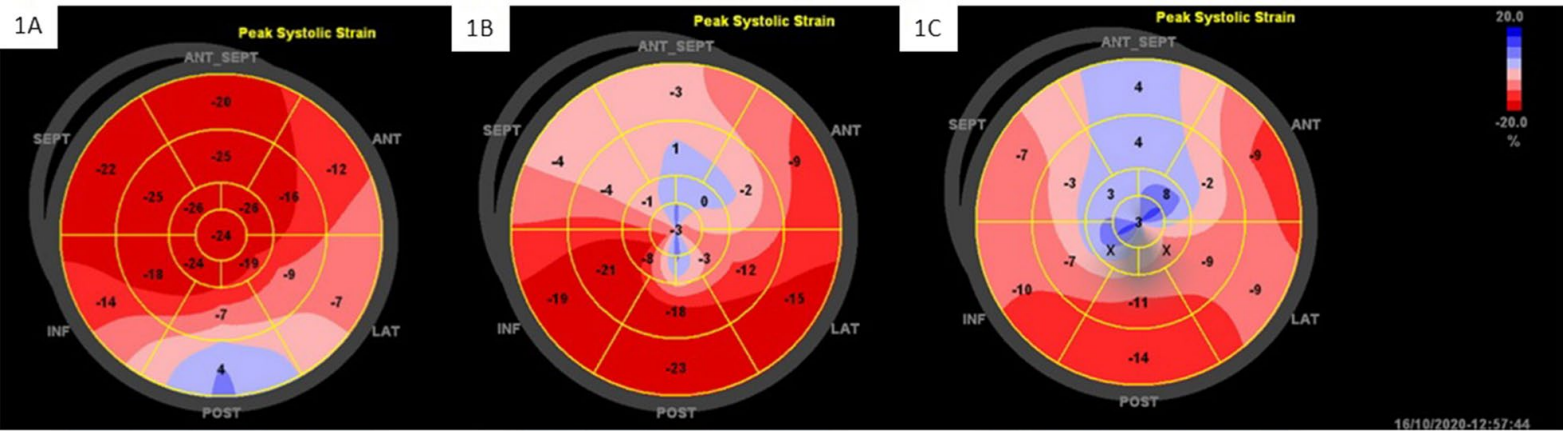
Examples of GLS. **A** Patient with normal LVEF (LVEF = 60.0%), GLS = − 17.3%; **B** patient with HFmrEF (LVEF = 43.0%), GLS = − 10.2%; **C** patient with HFrEF (LVEF = 35.6%), GLS = − 5.3%. GLS, global longitudinal strain; HFmrEF, heart failure with mildly reduced ejection fraction; HFrEF, heart failure with reduced ejection fraction LVEF, left ventricular ejection fraction

**Fig. 2:**
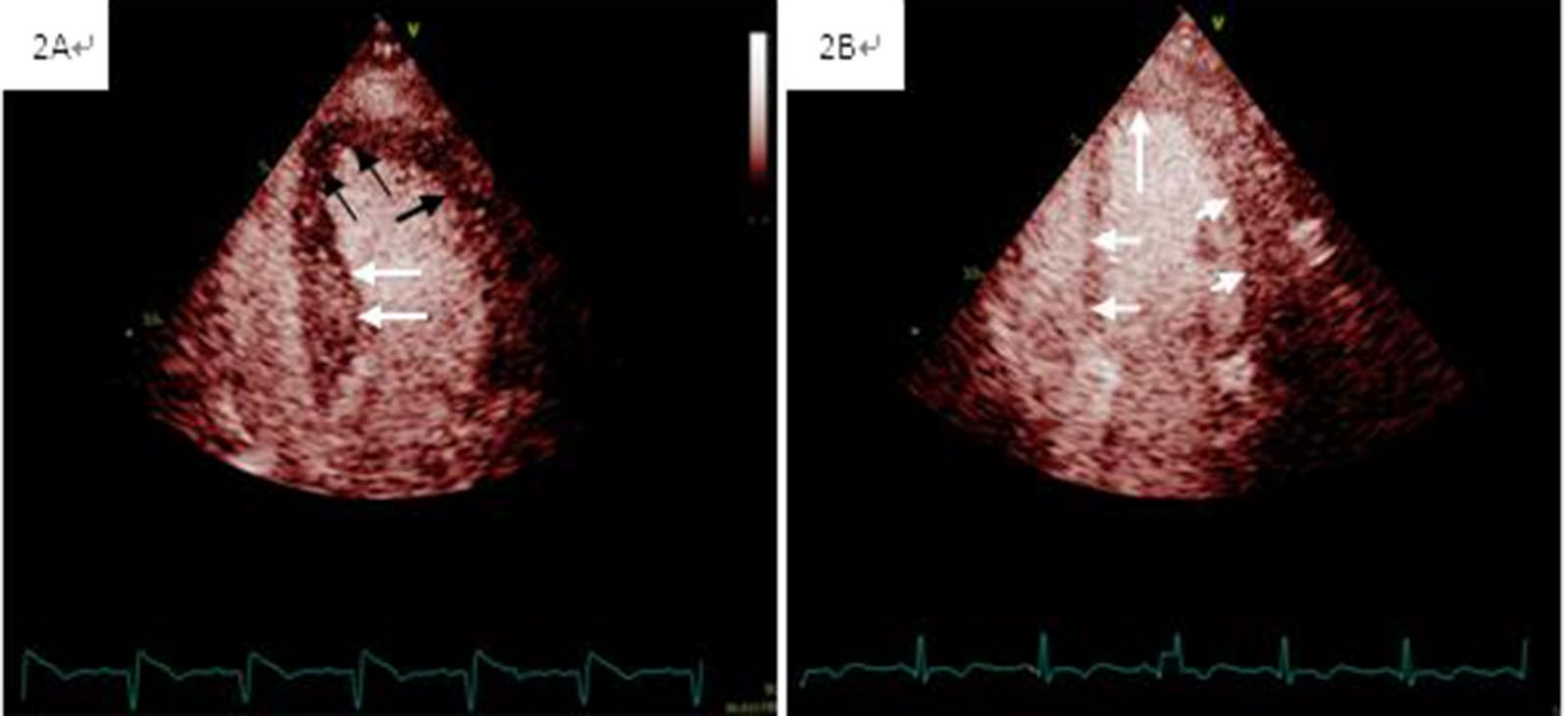
Examples of abnormal microvascular perfusion and normal microvascular perfusion. Demonstration of abnormal (**A**) and normal (**B**) myocardial contrast replenishment on real-time myocardial contrast echocardiograph. Black arrows: myocardial perfusion defect; white arrow: normal myocardial perfusion

All patients underwent echocardiography, STI, and MCE during hospitalization. Echocardiograms were performed using a Vivid E95 Console Ultrasound Machine (GE health care, USA). We used an M5Sc transducer with a 2.5–3.5 MHz imaging frequency for each study. At least three consecutive cardiac cycles were performed. According to the 2015 American Society of Echocardiography (ASE) and European Association of Cardiovascular Imaging (EACI) echocardiography guidelines [14], the internal dimensions were obtained using 2-dimensional (2D) echocardiography, and LVEFs and left ventricular end-diastolic volumes (LVEDVs) were measured using Simpson’s method. The left ventricular end-diastolic volumes index (LVEDVi) was obtained by correcting the LVEDV with the body surface area. The ASE 17-segment LV model was used to analyze regional WM; segments were scored as normal (score = 1), hypokinetic (score = 2), akinetic (score = 3), dyskinetic (score = 4), and ventricular aneurysm (score = 5), and the WM score index (WMSI) was derived as the average of the 17 segments. GLS measurements were made in three standard apical views (apical long axis, four-chamber, and two-chamber). Speckles were tracked frame-by-frame throughout the LV wall during the cardiac cycle, and basal, mid, and apical regions of interest were created. Segments that failed to track were manually adjusted by the operator, and GLS was calculated (Fig. 1). MCEs were performed in three-standard apical (four-, two-, and three chamber) views using sulfur hexafluoride (Sono-Vue) (Bracco International B.V.). Sulfur hexafluoride (Sono-Vue) was dissolved in saline (5 mL) and slowly injected intravenously (1 mL/min) with saline (5 mL) flushes. Chamber opacification and WM were observed in the left ventricular opacification mode, and myocardial perfusion was observed in the MCE mode. Real-time imaging was utilized with brief high mechanical index impulses to analyze replenishment. We also used a 17-segment model as described by the ASE/EACI [14] and points were allocated if the segment was completely replenished of contrast in the myocardium within 4 s (1 point), 4–10 s (2 points), and > 10 s (3 points). The myocardial perfusion index (MPI) was derived as the average of the 17 segments (Fig. 2) [15].

### Statistics

All statistical analyses were performed using the SPSS software (Version 21.0, IBM, Armonk, New York). Continuous variables are described as means ± standard deviations (SD) for normally distributed variables and as medians (interquartile ranges) for non-normally distributed variables. Categorical variables are described as numbers (percentages). Associations among variables were evaluated using analysis of variance (ANOVA) with LSD test, Kruskal–Wallis H test, and χ^2^ test. Statistical significance was set at *P* < 0.05.

## Results

### Baseline characteristics

Overall, 119 patients with STEMI were enrolled. In these patients, 32 patients (26.9%) had HFmrEF, 70 patients (58.8%) had normal LVEF (≥ 50%), and 17 patients (14.3%) had HFrEF. There were no differences in sex, age, BMI, smoking, clinical history, laboratory tests, and medicine treatment except for plasma B-type natriuretic peptide (BNP) levels (Table 1).

**Table 1:** Baseline characteristics of participants

Variable	Normal group (n = 70)	HFmrEF group (n = 32)	HFrEF group (n = 17)	*P* value
Female (%)	17 (24.3)	6 (18.8)	3(17.6)	0.741
Age, years	59 (51–69)	57 (47–68)	55 (44–64)	0.585
BMI, kg/ m^2^	24.9 ± 3.6	25.2 ± 3.9	26.5 ± 3.6	0.286
Smoker (%)	42 (60.0)	18 (56.3)	13 (76.5)	0.360
Hypertension (%)	43 (61.4)	23 (71.9)	9 (52.9)	0.388
Diabetes (%)	22 (31.4)	15 (46.9)	6 (35.3)	0.320
CKD (%)	6 (8.6)	5 (15.6)	2 (11.8)	0.566
AFL/Af (%)	7 (10.0)	3 (9.4)	2 (11.8)	0.965
WBC count, × 10^9^/L	9.1 (7.2–12.2)	9.8 (8.4–11.7)	9.3 (7.7–16.8)	0.480
Hemoglobin, g/ L	145 ± 16	147 ± 18	138 ± 20	0.199
PLT count, × 10^9^/L	223 (183–280)	261 (218–282)	240 (212–286)	0.102
FPG, mmol/L	5.9 (5.0–6.8)	5.6 (5.0–10.0)	6.8 (5.7–8.0)	0.162
HbA1c, %	6.6 ± 1.5	7.2 ± 1.7	6.7 ± 1.1	0.256
D-dimer, ng/mL	91 (58–159)	127 (53–343)	113 (67–264)	0.458
CRP, mg/L	4.7 (0.5–18.6)	9.9 (0.5–41.4)	11.2 (3.2–42.1)	0.363
BNP, pg/mL	151 (63–463)	290 (170–901)	625 (167–1534)	0.002
LDL-C, mmol/L	2.69 (2.21–3.62)	2.72 (2.41–3.20)	2.67 (2.09–3.47)	0.903
Triglyceride, mmol/L	1.33 (0.99–2.02)	1.38 (0.93–1.88)	1.57 (0.82–1.78)	0.872
Creatinine, μmol/L	78 (62–98)	79.5 (66.0–97.3)	87 (68–101)	0.593
Therapy				
Aspirin (%)	69(96.6)	31(96.9)	16(94.1)	0.557
Clopidogrel (%)	66(94.3)	32(100)	16(94.1)	0.383
Ticagrelor (%)	4(5.7)	0(0)	0(0)	0.235
ACEi (%)	34(48.6)	14(43.8)	4(23.5)	0.175
ARB (%)	15(21.4)	11(34.4)	8(47.1)	0.077
β-blocker(%)	55(78.6)	28(87.5)	15(88.2)	0.432
Statin (%)	69(98.6)	32(100)	17(100)	0.703
Trimetazidine (%)	3(4.3)	1(3.1)	1(5.9)	0.899

Data are expressed as mean ± SD, number (percentage), or median (interquartile range)

ACEi, angiotensin-converting enzyme inhibitor; AFL/Afib, atrial flutter/fibrillation; ARB, angiotensin II receptor blocker; BMI, body mass index; BNP, B-type natriuretic peptide; CKD, chronic kidney disease; CRP, C-reactive protein; FPG, fasting plasma glucose; HbA1c, glycosylated hemoglobin; HFmrEF, heart failure with mildly reduced ejection fraction; HFrEF, heart failure with reduced ejection fraction; LDL-C, low-density lipoprotein cholesterol; PLT, platelet; WBC, white blood cell

### STEMI data, coronary angiography findings and revascularization

Percutaneous coronary intervention (PCI) was performed in 110 patients (92.4%). Twenty-eight patients (23.5%) had received intravenous thrombolysis, and they subsequently underwent selected PCI. Nine patients did not receive any kind of revascularization (thrombolysis or PCI). The median time of pain to flow restored (including intravenous thrombolysis and percutaneous transluminal coronary angioplasty) was 5.3 (3.0–18.9) h. There were statistical differences in Killip grade, time of symptom to flow restored, culprit vessel, ST-segment elevation level, and time of ST-segment recovery > 50% of symptoms among the three groups. No statistical differences were observed in peak troponin I, thrombolysis percentage, PCI percentage, TIMI flow in the culprit vessel pre-PCI, and slow flow/no-reflow during PCI (Table 2).

**Table 2:** STEMI data, angiography findings and revascularization

Variable	Normal group (n = 70)	HFmrEF group (n = 32)	HFrEF group (n = 17)	*P* value
Killip grade				< 0.001
Grade I(%)	65 (92.9)	20 (62.5)	10 (58.8)	
Grade II (%)	4 (5.7)	9 (28.1)	3 (17.6)	
Grade III (%)	0 (0)	0 (0)	1 (5.9)	
Grade IV (%)	1 (1.4)	3 (9.4)	3 (17.6)	
Peak TnI, ng/ mL	42.0 (11.2–81.0)	72.3 (5.36–83.2)	65.7 (10.7–112.4)	0.811
Thrombolysis (%)	15 (21.4)	11 (34.4)	2 (11.8)	0.168
PCI (%)	67 (95.7)	30 (93.8)	13 (76.5)	0.064
No thrombolysis or PCI (%)	3 (4.3)	2 (6.3)	4 (23.5)	0.064
Time of symptom-to-flow restored, h	4.9 (2.5–10.8)	5.0 (3.0–24.4)	144.0 (5.3–264.0)	0.010
Culprit vessel				0.006
LAD (%)	33 (47.1)	24 (75.0)	13 (76.5)	
LCX (%)	10 (14.3)	1 (3.1)	0 (0)	
RCA (%)	27 (38.6)	5 (15.6)	3 (17.6)	
TIMI flow in culprit vessel pre-PCI < 3 (%)	40 (57.1)	15 (50.0)	10 (58.8)	0.314
Slow flow/no-reflow during PCI (%)	13 (18.6)	5 (15.6)	4 (23.5)	0.300
ECG parameters				
ST segment elevation level, mV	0.3 (0.2–0.4)	0.5 (0.3–0.6)	0.5 (0.2–0.5)	0.023
Time of STR > 50% to symptoms, h	8.1 (4.4–39.1)	13.8 (5.1–95.1)	96.0 (17.0–258.5)	0.001
Time of STR > 50% to PCI, h	0.7 (2.0–35.0)	2.6 (0.9–20.6)	22.1 (4.4–91.4)	0.171

Data are expressed as numbers (percentages) or medians (interquartile ranges)

HFmrEF, heart failure with mildly reduced ejection fraction; HFrEF, heart failure with reduced ejection fraction; LAD, left anterior descending artery; LCX, left circumflex coronary artery; PCI, percutaneous coronary intervention; STEMI, ST-segment elevation myocardial infarction; TnI, troponin I; RCA, right coronary artery; STR, ST-segment recovery

### Echocardiogram, STI and MCE

All the patients had completed echocardiogram and MCE within 7 days after STEMI. One hundred and ten (92.4%) patients had completed PCI before MCE. The rates of PCI before MCE of the 3 groups were showed in Table 2 and no significant difference was observed among the patients with normal LVEF, HFmrEF and HFrEF. The mean GLS of all 119 patients was − 11.9 ± 3.8%. Fourteen patients (11.8%) had a GLS above − 18.0%, and seven patients (5.9%) were within normal limits when the cut-off of GLS was set at − 20.0%. The mean LV end-diastolic dimension was 4.9 ± 0.6 cm. The median LVEF was 54.0 (44.6–61.5)%. The median WMSI was 1.47 (1.25–1.75). Median GLS was − 11.3 (− 9.0 to − 14.3)%. One hundred and seven patients (90.1%) exhibited regional WM abnormalities. Ventricular aneurysm was found in 18 patients (15.1%) and LV thrombosis in seven patients (5.9%). Seventy-seven patients (64.7%) exhibited an abnormal MPI (> 1.00), which suggested CMD.

**Fig. 3:**
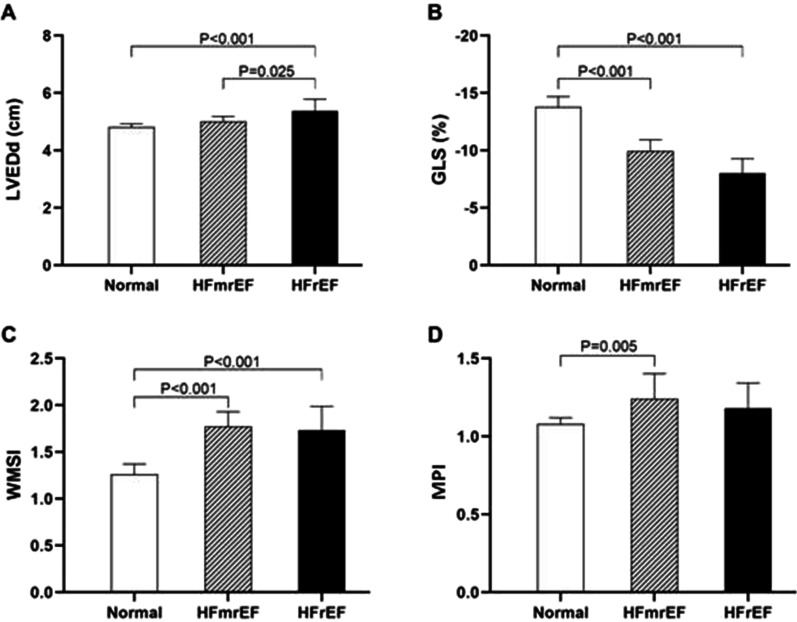
STI and MCE parameters among the groups. Comparison of LVEDd (**A**), GLS (**B**), WMSI (**C**), and MPI (**D**). GLS, global longitudinal strain; left ventricular end-diastolic dimension; MCE, myocardial contrast echocardiography; MPI, myocardial perfusion index; STI, speckle tracking imaging; WMSI, wall motion score index; LVEDd, left ventricular end-diastolic dimension

All the above parameters were significantly different among the three groups (Table 3). The GLS of the HFmrEF group was not significantly different from that of the HFrEF group (*P* = 0.052), but they were both lower than that of the normal group (*P* < 0.001). The WMSI in the HFmrEF group was 1.76 ± 0.30, which was comparable to that in the HFrEF (1.81 ± 0.43, *P* = 0.618), but poorer than that in the normal group (1.33 ± 0.25, *P* < 0.001) (Fig. 2). The incidence of CMD (MPI > 1) in the HFrEF, HFmrEF, and normal groups was 88.2%, 75.0%, and 54.3%, respectively (*P* = 0.011). Patients with HFmrEF displayed a mean MPI of 1.24 ± 0.33, which was not significantly different from that in the HFrEF group (1.18 ± 0.19, *P* = 0.486), but was significantly poorer than that in the normal group (1.08 ± 0.14, *P* = 0.005) (Fig. 3). Meanwhile, there was a significant difference in the incidence of LV thrombosis among the HFrEF, HFmrEF, and normal groups (17.6%, 9.4%, and 1.4%, respectively, *P* = 0.024).

**Table 3:** Echocardiogram, STI and MCE parameters among the groups

Variable	Normal group (n = 70)	HFmrEF group (n = 32)	HFrEF group (n = 17)	*P* value
LVEDd, cm	4.8 ± 0.5	5.0 ± 0.5	5.4 ± 0.8	0.001
LVEDV(ml)	101(79–119)	114(95–133)	140(115–189)	< 0.001
LVEDVi (ml/m^2^)	56(46–63)	61(52–72)	76(66–103)	< 0.001
LVEF, %	61.0 ± 7.4	45.4 ± 2.6	32.1 ± 5.8	< 0.001
GLS, %	− 13.8 ± 3.5	− 9.9 ± 2.5	− 8.0 ± 2.3	< 0.001
RWMA (%)	58 (82.9)	32 (100)	17 (100)	0.009
Ventricular aneurysm (%)	5 (7.1)	7 (21.9)	6 (35.3)	0.007
WMSI	1.33 ± 0.25	1.76 ± 0.30	1.81 ± 0.43	< 0.001
MPI	1.08 ± 0.14	1.24 ± 0.33	1.18 ± 0.19	0.015

Data are expressed as mean ± SD or number (percentage)

GLS, global longitudinal strain; LVEDd, left ventricular end-diastolic dimension; LVEDV, left ventricular end-diasolic volume; LVEDVi, left ventricular end-diasolic volume index; LVEF, left ventricular ejection fraction; MPI, myocardial perfusion index; STI, speckle tracking imaging; MCE, myocardial contrast echocardiography; RWMA, regional wall motion abnormality; WMSI, wall motion score index

## Discussion

Currently, the therapeutically relevant classification of HF remains based on LVEF. All guidelines use the terminology of HFpEF and HFrEF, but differ in the terminology used in patients with EFs between 40 and 49% [8]. HFpEF is a heterogeneous syndrome with various clinical presentations. Compared with patients with LVEF > 50%, those with LVEF 40–50% exhibit a higher prevalence of coronary heart disease and may have similar characteristics to those with LVEF < 40% [16]. Patients with HFmrEF may show similar responses to treatment as those with LVEF < 40% [5]. A post-hoc analysis of the TOPCAT trial suggested that mineralocorticoid receptor antagonists reduce morbidity and mortality in elderly patients with HF with a homogenous treatment effect in HFpEF [4, 5]. The PARADIGM-HF trial demonstrated that angiotensin receptor-neprilysin inhibitors (ARNis) improved survival in patients with HFrEF, but failed to reduce the incidence of the primary endpoint in patients with HFpEF. However, subgroup analysis suggested that the effect of ARNis on the primary endpoints was significant in patients with HFpEF with lower LVEF [6]. Therefore, the 2021 ESC Universal Definition and Classification of HF proposed HFmrEF as the category for patients with an LVEF between 40 and 49% [8]. Further classification into HFmrEF has potential utility as well as challenges due to its ambiguity, uncertainty, and dynamic trajectory. Patients with HF stratified according to different categories of LVEF present diverse phenotypes of demography, clinical presentation, etiology, and outcomes. Patients with HFmrEF possess similar features as those with HFrEF, including age, sex, and ischemic etiology [17], but have better outcomes, as opposed to higher rates of mortality and HF readmissions seen in patients with HFrEF [18]. The ‘gray zone’ of LVEF between 40 and 50% requires further characterization. STEMI is an important cause of HF, and the state of LV function after STEMI is an important indicator of prognosis. GLS derived from 2D-STE can detect LV systolic dysfunction at an earlier stage than LVEF and is associated with a worse outcome [19, 20]. MCE can be used for CMD and provides important information for prognosis. Therefore, we evaluated LV function and coronary microcirculation in patients with mildly reduced LVEF after STEMI using 2D-STE and MCE.

A total of 119 patients with STEMI were enrolled in this study, 26.9% with HFmrEF and 14.3% with HFrEF. The median BNP levels in the HFmrEF and HFrEF groups were 290 (170–901) pg/mL and 625 (167–1534) pg/mL, respectively. A total of 58.8% of the patients had an LVEF ≥ 50%. The normal value for GLS depends on the definition of the measurement position in the myocardium, vendor, and version of the analysis software, resulting in considerable heterogeneity in the published literature. The 2015 ASE guidelines recommend that a peak GLS in the range of -20% can be expected in a healthy person [14]. In our study, although 58.8% of patients had a normal LVEF (≥ 50%), most patients had impaired GLS. Only 5.9% of patients had a GLS above − 20.0%, 88.2% patients had a GLS below -18.0%, and the median GLS of all patients was − 11.9 ± 3.8%. This means that GLS can detect LV systolic dysfunction earlier than LVEF. Doeblin et al. observed that patients with HFmrEF differed from healthy individuals and shared similarities with patients with HFrEF in cardiac magnetic resonance parameters of fibrosis and inflammation [21]. By means of STI, we found that the GLS in patients with HFmrEF was − 9.9 ± 2.5%, which was more comparable to that in patients with HFrEF (− 8.0 ± 2.3%, *P* = 0.052), but significantly lower than that in the normal group (− 13.8 ± 3.5%, *P* < 0.001). Additionally, WMSI in the HFmrEF group also resembled that in the HFrEF group (1.76 ± 0.30 vs. 1.81 ± 0.43, *P* = 0.618) and was significantly poorer than that in the normal group (1.33 ± 0.25, *P* < 0.001). These results reveal significant adverse remodeling beyond systolic functional impairment and WM in patients with HFmrEF, comparable to the changes seen in patients with HFrEF, but different from those in patients with LVEF > 50%. And these distinctions were not affected by the medicine treatment. Therefore, patients with HFmrEF after STEMI require greater attention.

Despite successful opening of the culprit epicardial vessel, adverse LV remodeling, HF, and even death still occur. CMD, as a possible reason, has attracted more attention in recent years [22]. Studies have shown that the prevalence of CMD is 60–89% in successfully treated STEMI [12, 22]. We detected 119 patients with STEMI by MCE and identified 66.7% of patients with CMD. This result was consistent with previous literature. Furthermore, we found no significant difference in MPI between patients with HFmrEF and patients HFrEF (1.24 ± 0.33 vs 1.18 ± 0.19, *P* = 0.486), which were both poorer than that in normal group (1.08 ± 0.14, *P* = 0.005). This result reveals that the coronary microcirculation status in patients with HFmrEF is comparable to that in patients with HFrEF, but poorer than that in patients with LVEF > 50%. The presence of CMD after STEMI predicts adverse prognoses such as rehospitalization, HF, and mortality [23, 24]. It is also a powerful predictor of LV adverse remodeling [25]. The presence and extent of CMD after primary PCI in STEMI are strongly associated with major adverse cardiovascular events within 1 year [11]. The occurrence of CMD in patients with HFmrEF after STEMI requires greater attention, and its effect on long-term prognosis requires further study. It was unexpected that the MPI in the HFrEF group was higher than that in the normal group, but no statistical difference was observed between the two groups. This may be due to the small sample size, and thus, further research is required.

HFmrEF is a new concept. Actually, it is still a controversial topic whether HFmrEF pertinently addresses a distinct category or merely a transition zone between HFpEF and HFrEF. Whereas it’s been well accepted that HFrEF and HFpEF differed in terms of the underlying aetiologies, demographics clinical feather, and morbidities. Conversely, it’s still not clear when comes to the HFmrEF patients, despite the evolving knowledge and recognition in HFmrEF.We described the UCG phenotypic characteristics of patients with HFmrEF. According to our knowledge, it’s the first time to view HFmrEF in the sight of LV systolic function and microcirculation. The significance of our finding is the GLS and MPI of HFmrEF patients were similar to thse of the HFrEF ones but not the normal ones, i.e. it’s plausible to construe HFmrEF as sort of HFrEF at least in one sense. Since HFmrEF is pretty new and evolving, controversy haunts doctors about whether HFmrEF should be considered a distinct category or a transition zone. Our encouraging finding provides the evidence to help give an end to this controversial topic.

Patients with HFrEF suffer functional, structural, cellular, and interstitial changes that are known as left ventricular remodeling. The treatments targeting remodeling are a mainstay of HFrEF therapy. Conversely, these treatments failed to benefit the patients with HFpEF. However, it’s intriguing that no solid conclusion could be reached for the HFmrEF ones given the dearth of understanding of HFmrEF. We demonstrated that HFmrEF is more analogous to the HFrEF but the normal one. That hinted at the plausible value of positive treatments. Further, our available data suggested a possible benefit from treatment against remodeling.

In our study, the time of symptom-to-flow restoration in the HFrEF group [144 (5.3–264.0) h] was significantly longer than that in the normal group [4.9 (2.5–10.8) h] and HFmrEF group [5.0 (3.0–24.4) h]. Indeed, patients with HFrEF had worse clinical symptoms, critical conditions, and delayed PCI combined to develop worse cardiac function in this group. Therefore, delayed PCI resulted in patients with HFrEF experiencing even worse LV systolic dysfunction and CMD. Nevertheless, patients with HFmrEF showed similar LV systolic function and coronary microcirculation perfusion to those with HFrEF, and these results further confirmed that patients with HFmrEF might have a poor prognosis. However, our study only involved patients with STEMI who underwent either emergency PCI, selective PCI (including post-thrombolysis), or patients who did not receive any revascularization (thrombolysis or PCI). Meanwhile, the study is relatively small and includes only 119 patients. It’s the limit of this study. However, we demonstrated the difference among the groups in spite of the small size. In the future, further studies with larger sample sizes are required. Meanwhile the lack of clinical outcome and follow-up data is the limitation of our article. Whether echocardiographic parameters will recover during follow-up and whether GLS will improve earlier than LVEF? More study about the prognosis and echocardiographic parameters outcome need to be done in the future.

## Conclusions

GLS derived from STI can detect LV systolic dysfunction earlier than LVEF. LV systolic function, coronary microcirculation perfusion, and WM abnormalities associated with HFmrEF after STEMI are all different from those in normal LVEF (≥ 50%), but comparable to those associated with HFrEF. Therefore, patients with HFmrEF after STEMI require urgent attention and more appropriate management.

## Data Availability

The datasets used and analyzed during the current study are available from the corresponding author on reasonable request.

## Abbreviations

ACEi: Angiotensin-converting enzyme inhibitor
AFL/Afib: Atrial flutter/fibrillation
ARB: Angiotensin II receptor blocker
BMI: Body mass index
BNP: B-type natriuretic peptide
CKD: Chronic kidney disease
CRP: C-reactive protein
EF: Ejection fraction
FPG: Fasting plasma glucose
GLS: Global longitudinal strain
HbA1c: Glycosylated hemoglobin
HFmrEF: Heart failure with mildly reduced ejection fraction
HFrEF: Heart failure with reduced ejection fraction
LDL-C: Low-density lipoprotein cholesterol
LVEDd: Left ventricular end-diastolic dimension
LVEDV: Left ventricular end-diasolic volume
LVEDVi: Left ventricular end-diasolic volume index
LVEF: Left ventricular ejection fraction
MCE: Myocardial contrast echocardiography
MPI: Myocardial perfusion index
PLT: Platelet
PCI: Percutaneous coronary intervention
LAD: Left anterior descending artery
LCX: Left circumflex coronary artery
RCA: Right coronary artery
RWMA: Regional wall motion abnormality
STEMI: ST-segment elevation myocardial infarction
STI: Speckle tracking imaging
STR: ST-segment recovery
TnI: Troponin I
WBC: White blood cell
WMSI: Wall motion score index
