# Correction: Left ventricular function and coronary microcirculation in patients with mild reduced ejection fraction after STEMI

**DOI:** 10.1186/s12872-022-02917-x

**Published:** 2022-11-14

**Authors:** Yuliang Ma, Lan Wang, Wenying Jin, Tiangang Zhu, Jian Liu, Hong Zhao, Jing Wang, Mingyu Lu, Chengfu Cao, Bailin Jiang

**Affiliations:** 1 Beijing Key Laboratory of Early Prediction and Intervention of Acute Myocardial Infarction; Center for Cardiovascular Translational Research, Department of Cardiology, Peking University People’s Hospital, Beijing, China; 2 Department of Anesthesiology and Pain Medicine, Peking University People’s Hospital, Beijing, China

**Correction: BMC Cardiovasc Disord 22, 423 (2022)**

**https://doi.org/10.1186/s12872-022-02846-9**

An author name ‘Yuliang Ma’ has been inserted in the author by line. So, the text will read as follow:

Yuliang Ma, Lan Wang and Wenying Jin contributed equally to this work

The original article [1] has been corrected.
